# Supplementary figures and images for: The MorbidGenes panel: a monthly updated list of diagnostically relevant rare disease genes derived from diverse sources
Source: Hum Genet. 2024 Oct 28;143(12):1459–63. doi: 10.1007/s00439-024-02711-z (PMC11576763; doi:10.1007/s00439-024-02711-z)

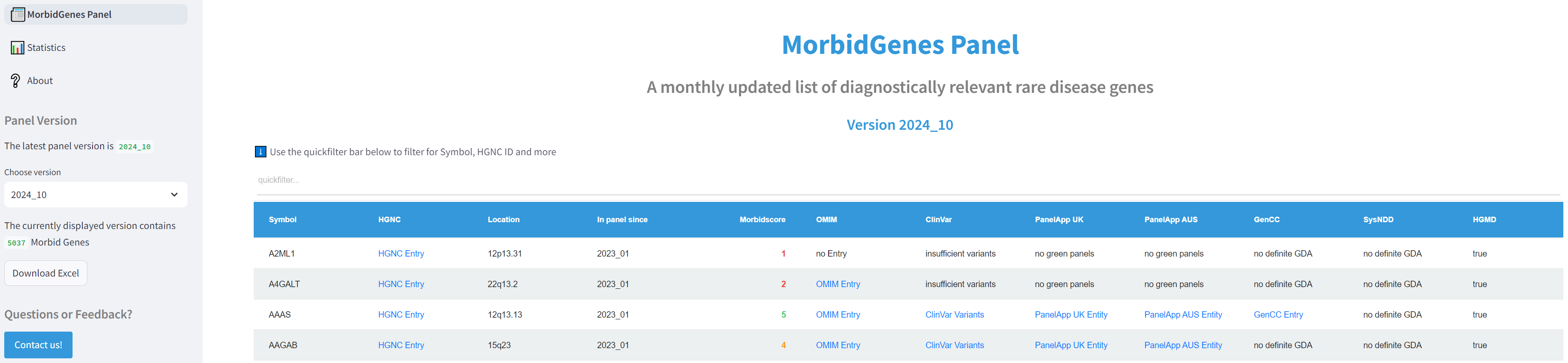

Supplement: Supplementary file 1 — Supplementary Material 1: Fig. 1. Screenshot of https://morbidgenes.uni-leipzig.de. The web interface provides a dropdown menu to filter for specific panel versions and a download button for the displayed panel. The grid interface contains links to the respective data sources. The navigation bar on the left side leads to two additional pages: “Statistics”, which provides monthly updated plots comparable to Fig. 1; and “About”, which provides additional background information, limitations of the panel as well as the legal notice. [file 439_2024_2711_MOESM1_ESM.png]
